# Safety and Efficacy of Nontuberculous Mycobacteria Treatment among Elderly Patients

**DOI:** 10.3390/medicina56100517

**Published:** 2020-10-02

**Authors:** Yoshitaka Uchida, Jiro Terada, Tetsuya Homma, Hatsuko Mikuni, Kuniaki Hirai, Haruhisa Saito, Ryoichi Honda, Hironori Sagara

**Affiliations:** 1 Department of Medicine, Division of Respiratory Medicine and Allergology, Showa University School of Medicine, Tokyo 142-8555, Japan; for.u.goodlife@gmail.com (Y.U.); ha.mikuni392@gmail.com (H.M.); medi123@infoseek.jp (K.H.); sagarah@med.showa-u.ac.jp (H.S.); 2 Department of Respirology, Asahi General Hospital, Chiba 289-2511, Japan; jirotera@chiba-u.jp (J.T.); hasaito@hospital.asahi.chiba.jp (H.S.); honda@sapmed.ac.jp (R.H.); 3 Department of Respirology, Graduate School of Medicine, Chiba University, Chiba 260-8670, Japan

**Keywords:** elderly, nontuberculous mycobacterium, NTM, treatment, safety

## Abstract

*Background and objectives*: Incidence rates of pulmonary nontuberculous mycobacterial (NTM) disease have been increasing, especially in the elderly population. Given the limited evidence regarding the safety and efficacy of NTM treatment, this study aimed to evaluate the same among elderly patients. *Material and methods*: Patients diagnosed with NTM disease at a tertiary hospital from January 2007 to December 2017 were enrolled and data were then retrospectively collected. Data of elderly patients who received antimycobacterial treatment were then analyzed. *Results*: A total of 161 patients satisfied the diagnostic criteria for NTM disease. There were 40 elderly patients who received treatments. Of the patients, 60% received the guideline oriented standard regimens. Single drug regimens were administered to 22.5% of patients. Only 55.0% of the patients were able to continue any treatment. Treatment-related discontinuation was observed in 44.4% of discontinued or changed patients. There were no significant differences in the characteristics of patients with or without adverse events. Patients who were able to continue the treatment for >12 months had a lower proportion of activities of daily living (ADL) disability (nine in 18, 50.0% vs. three in 22, 13.6%, *p* = 0.018) and heart disease (six in 18, 33.3% vs. 1/22, 4.6%, *p* = 0.033). Sputum culture conversion was achieved in 28 out of 40 (70.0%) elderly patients treated, and those who achieved sputum culture conversion had more standard regimens prescribed than those who failed sputum culture conversion (21 in 28, 75% vs. 3 in 12, 25%; *p* = 0.005). *Conclusion*: Age may not be an obstacle for receiving the benefits of the treatment of NTM disease with a precise evaluation of patient’s comorbidities. Furthermore, elderly patients without heart disease and ADL disability may have better rate of continuing the NTM treatment. The current study suggested that selecting standard regimens to treat pulmonary NTM is important for elderly patients.

## 1. Introduction

The prevalence of pulmonary nontuberculous mycobacterial (NTM) disease has been increasing over the past few decades with varying rates in different parts of the world. Previous reports observed a sharp increase in the incidence rate of NTM disease in Japan (14.1 per 100,000 person-years in 2014, approximately 2.6 times higher than 2007) [1]. One study showed that the incidence and prevalence rates of NTM diseases in Japan were higher compared to other countries in North America, Europe, and Oceania [2]. Moreover, another study showed that among Japanese patients with NTM disease, 54.4%–86.6% were over 70 years old [3,4], which appeared to be much higher than in other countries [5]. Elderly patients are at risk for NTM-related mortality and disease progression [6,7]. In addition, elderly patients generally tend to have several and complicated comorbidities, which are considered one of the important predictors of death [8].

However, detailed information regarding elderly individuals with NTM disease is scarce. Previous large studies on NTM treatment cited by the guidelines [9] included elderly patients, but such patients’ mean age was 49.5–75.5 [10,11,12,13,14,15,16,17,18,19,20,21,22,23]. Therefore, safety and efficacy of NTM disease treatment among elderly patients is still limited. This may lead to unrecommended treatment modifications, such as decreasing the number of antibiotics to single drug regimens [6,9,24]. Given the lack of evidence regarding the safety and efficacy of NTM treatment and the high prevalence of comorbidities in elderly patients, the present study aimed to evaluate the safety and efficacy of NTM treatment among elderly patients pulmonary NTM infection.

## 2. Materials and Methods

This retrospective study was conducted at Asahi General Hospital, a tertiary hospital in Japan. The study protocol was approved by the institutional ethics review committee (approval number 2018032023) and performed in accordance with the amended Declaration of Helsinki. Written informed consent was given and personal information was anonymized. Patients diagnosed with NTM disease were enrolled and analyzed.

The study included 181 patients whose age was above 20 years old with at least independent two positive cultures from expectorated sputum specimens or one positive culture from bronchial wash or bronchoalveolar lavage fluid specimens between January 2007 to December 2017. Patients with known HIV infection, cystic fibrosis, or who did not satisfy the following diagnostic criteria were excluded. The diagnosis was based on the 2007 American Thoracic Society criteria [25], which included the following: (1) symptoms of a pulmonary disease and exclusion of other lung diseases; (2) nodular or cavitary opacifications on chest radiograph or multifocal bronchiectasis with multiple small nodules on high-resolution computed tomography (HRCT) scan; and (3) at least two positive cultures from separately expectorated sputum specimens or one positive culture from bronchial wash or bronchoalveolar lavage (BAL) fluid. Among the 181 patients with positive culture, 161 met the diagnosis criteria. The patients who were below 70 years old was also excluded and age of 70 years old and above were analyzed. Although many other countries have defined elderly individuals as those aging ≥ 5 years old, considering past study reporting many of Japanese patients with NTM disease are over 70 years old [3,4], we analyzed the patients who were 70 years old or more. We reviewed the clinical information, laboratory data including sputum culture, radiological findings, and used antibacterial regimens with AE information from 40 patients who were administrated the antibiotics to treat NTM disease.

Incidence of continuation of treatment, and adverse events (AEs) were determined from the medical records. Activities of daily living (ADL) disability was defined as the use of a wheelchair, any use of nursing care insurance, or any need for care with daily living recorded by any medical staff, including the care manager. The habit of alcohol was defined by at least three days or more per week of alcohol consumption with any amount. Respiratory diseases (asthma, chronic obstructive pulmonary disease (COPD), pulmonary fibrosis, etc.), malignant diseases, and heart diseases (heart failure, ischemic heart disease, arrythmia, etc.) were recorded to characterize patient’s comorbidities. Chest radiological responses was assessed by chest radiograph or high-resolution computed tomography (HRCT), and assessment of chest radiograph and HRCT images were performed by at least one respiratory physician and one radiologist. Sputum culture conversion rates were also evaluated. When sputum specimens were not available, sputum culture conversion were marked as “improved”. “Prescribed regimens” are defined as an antibiotic regimen that were used constantly. Data of changes of initial regimen to other regimen, treatment discontinuation, dosage adjustment, or regimen modification were collected. The duration of treatment was defined by the British Thoracic Society Guideline [9]. Patients who received at least 12 months of treatment were recorded as “continued”, those who adjusted dosage or modified their regimens and were treated for at least 12 months were recorded as “changed”, and those who were unable to continue their treatment for 12 months were recorded as “discontinued”. Standard regimens were defined as combination of rifampicin (RFP), ethambutol (EB), and clarithromycin (CAM) for *M. avium* complex, combination of isoniazid (INH), RFP, and EB for *M. kansasii*, and combination of amikacin (AMK), CAM, and imipenem/cilastatin (IPM/CS) for *M. abscessus.*

Fisher’s exact tests or Pearson’s chi-squared statistic were used to compare group frequencies for categorical variables, while the Wilcoxon rank-sum test was used to compare group means for continuous variables. Significance was determined if P value was less than 0.05. All analyses were performed using JMP^®^ Pro 14.0.0 statistical software (SAS Institute Inc., Cary, NC, USA).

## 3. Results

### 3.1. Study Population and Characteristics

The baseline characteristics of treated elderly patients with NTM are described in Table 1. Mean age was 79.1 years old. Any comorbidities such as respiratory disease, malignancy, and heart disease were seen in 24/40 (60%) patients. The most popular symptoms were cough (n = 23, 57.5%) and sputum (n = 20, 50%). There were 9/40 (22.5%) patients were prescribed only one drug and three out of 40 (7.5%) patients were prescribed two drugs. Single drug regimens, which included erythromycin (EM), levofloxacin (LVFX), and CAM, were prescribed to five, one, and three elderly patients, respectively. Two drug regimens included EB + CAM, AMK + LVFX, and CAM + LVFX, each of which were prescribed to one elderly patient, respectively. Regimens with three or more drugs prescribed to elderly patients included RFP + EB + CAM (n = 22), INH + RFP + EB (n = 1), IPM/CS + Tobramycin + CAM (n = 1), Faropenem (FRPM) + RFP + LVFX +CAM (n = 1), EB + CAM + LVFX (n = 1), CAM + FRPM + Sitafloxacin (n = 1), AMK + CAM + IPM/CS (n = 1).

**Figure 1:**
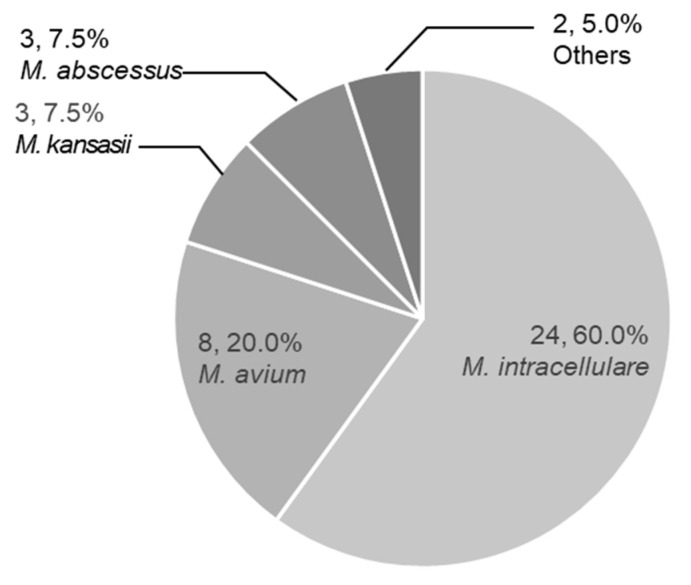
Microbiological findings. Etiology of treated elderly patients. Numbers of patients and percentages are shown.

Most patients were infected either with *M. intracellulare* (n = 24), or *M. avium* (n = 8), which accounted for 80% of patients (Figure 1). Other species included *M. kansasii* (n = 3), *M. abscessus* (n = 3), *M. chelonae* (n = 1), and rapidly growing mycobacteria, which was not able to be determined (n = 1).

### 3.2. Continuation of Treatment

**Figure 2:**
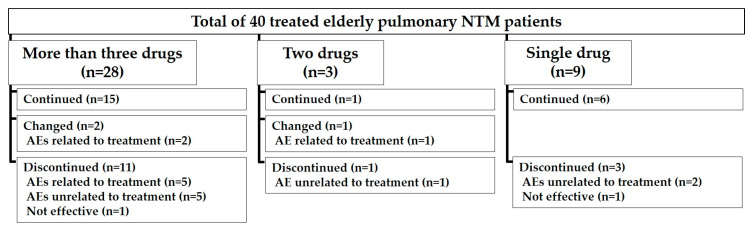
Overview of the number of patients according to the number of antibiotics used in the treatment. Furthermore, the patients were divided to treatment continued, changed, or discontinued. Abbreviations: NTM = nontuberculous mycobacterium; AE: adverse event.

Treatment was continued for 12 months in 22/40 (55%) patients. Specifically, 15/28 (53.6%), 1/3 (33.3%), and 6/9 (66.7%) patients were able to continue regimens with three or more drugs, two drugs, and single drug respectively (Figure 2). Discontinuation due to treatment-related AEs were observed in 8/18 (44.4%) patients of all discontinued or changed patients. A comparison of the characteristics between treated elderly patients who changed or discontinued the treatment (n = 18) and those who were able to continue their treatment (n = 22) is presented in Table 2. Patients who changed or discontinued the treatment comprised a greater proportion of patients with ADL disability (nine in 18, 50.0% vs. three in 22, 13.6%, *p* = 0.018) and heart disease (six in 18, 33.3% vs. one in 22, 4.6%, *p* = 0.033). Those six patients with heart disease had to discontinue their treatment either due to treatment related AEs (one in six patients) or decline of general status (five in six patients). Nine patients with ADL disability who changed or discontinued their treatment had to discontinue the treatment for treatment related AEs (six in nine patients), decline of general status (two in nine patients), or death by that was not related to NTM infection (one in nine patients).

### 3.3. Adverse Events Associated with Treatment

A comparison of the characteristics between patients with AEs associated with treatment and those without AE is presented in Table 3. AEs associated with NTM treatment were observed in 19 in 40 (47.5%) patients. The most common AE was gastrointestinal symptoms (13 in 40, 32.5%) and skin rash (five in 40, 12.5%). When AMK or tobramycin were prescribed, therapeutic drug monitoring (TDM) was performed and all patients who took AMK or tobramycin were in the safe range. Also, electrocardiogram (ECG) was monitored occasionally and prolonged QT interval was not detected in any patients. There were no significant differences in characteristics.

### 3.4. Efficacy

A comparison of the characteristics between patients who failed and those who achieved sputum culture conversion is presented in Table 4. Sputum culture conversion was achieved in 28/40 (70.0%) patients. Patients who achieved sputum culture conversion were more on standard regimens than patients who failed sputum culture conversion (21 in 28, 75.0% vs. three in 12, 25.0%; *p* = 0.005). Also, hemoptysis was less seen in patients who achieved sputum culture conversion (four in 28, 14.3% vs. six in 12, 50.0%; *p* = 0.041).

A comparison of the characteristics between patients with and without radiographic improvement is presented in Table 5. Radiographic findings improved in 23 out of 40 (57.5%) patients. Although patients who had a smoking history appeared more in the improved group (11 in 23, 47.8% vs. three in 17, 17.6%; *p* = 0.038), the same group were taking more standard regimens prescribed than patients who had no or worse radiographic changes (17 in 23, 73.9% vs. 7 in 17, 41.2%; *p* = 0.037). Also, it was suggested that, if standard regimens were prescribed, nodular bronchiectatic radiological type seemed to have better outcome (18 in 23, 78.3% vs. 8 in 17, 47.1%).

## 4. Discussion

This study showed that the continuation of NTM treatment among the elderly was dependent on ADL condition and heart disease comorbidity. Secondly, the prescription of standard regimen was related to achieving sputum culture conversion and better outcome of radiological findings. Importantly, about 30% of elderly patients were administered with single to two drug regimens which were not recommended by the current guidelines [9,25,26].

The presence of heart disease, such as chronic heart failure, previous myocardial infarction, and arrythmia were related to discontinuation of NTM treatment among our study patients. In a previous study, death due to NTM progression occurred in only 14.6% of patients, and the main cause of death was non-pulmonary disease (53.8%) [6]. Along this line, numbers of comorbidities were considered to be an important predictor of death [8]. The main causes of death among NTM patients was heart disease and it was reported to be between 12.5% and 30% [18,21]. Also, others have reported that heart disease was the main risk of mortality [27]. Although, previous reports have not precisely focused on relationship between treatment discontinuation and patient’s comorbidities, our current study showed heart disease was related to discontinuation of the treatment, which was novelty of this study [26]. Also, it seemed to be important to continue the standard regimen since the culture conversion and radiological improvement were achieved more in those group in the current study. This study also confirmed that patients who changed or discontinued the treatment had more comorbidity of heart disease. Therefore, comorbidity of heart disease in elderly patients maybe one of the predictors of treatment discontinuation in elderly NTM patients.

ADL disability was significantly observed in patients who had to change or discontinue the initial treatment in this study. Thus, in addition to cardiac comorbidities, ADL disability may serve as a predictor of treatment discontinuation. The older population tend to have degrees of disability and this study adds ADL evaluation is important when treating elder NTM patients, since ADL disability was not discussed in previous NTM studies. The only past study reported related to ADL was the relationship between spine muscles, health-related QOL (HRQL), and prognostic physiological parameters, such as BMI and pulmonary function among NTM infected patients [28]. ADL disability was a risk factor for not only death, but treatment-related AEs when treating pulmonary tuberculosis [29,30]. Along with choosing the effective drug regimen, nutrition and physical activity care seems to be important when treating elderly patients, since malnutrition and frailty were highly related to loss of ADL in such group [31,32,33]. According to previous study, non-drug treatment, such as nutrition therapy or physical rehabilitation, was useful when treating elderly patients with NTM disease [31]. Background characteristics should be carefully evaluated when treating elder population group and ADL should be more cared.

Sputum culture conversion was observed among patients who received a guideline-oriented standard regimen in our current study. Elderly patients have comparable efficacy to past reports with young population [10,11,12,13,14,15,16,17,18,19,20,21,22,23]. Similarly, this study indicated that radiological improvement was also observed among patients who received standard regimen. This observation was consistent with sputum culture conversion for NTM and this is supported by past study [34]. Thus, the efficacy of treatment might be limited when prescribing modified regimens, such as single or two drug regimens. Age might not be an obstacle to prescribe the guideline recommended standard regimens in the elderly patients. Further study is warranted to clarify this certain clinical question.

The use of multiple antibiotic regimens for elderly patients, who is over 70 years old, is not still well studied [10]. Several previous studies reported usefulness of two-drug regimen, but comparison to standard regimen was lacked [35,36]. Moreover, there were studies that reported 12.6% of bronchiectatic MAC pulmonary infections were treated with monotherapy and showed some efficacy [6]. These lines of evidence led physicians to use fewer drugs for pulmonary NTM infection treatment among elderly patients due to absence of sufficient clinical study. However, modified therapies, especially macrolide monotherapy is not recommended [24]. In this study, more than half of the patients were able to take regimens with three or more drugs without any AEs, suggesting a better outcome for those patients.

Given that the present study has methodological limitations related to its retrospective nature and this study was conducted in single center. Although we were able to enroll 181 patients, it may raise the risk of bias due to the small number of participants who were finally analyzed. A multi-center prospective study is warranted to clarify the determination of patients eligible for standard regimens among elderly patients with pulmonary NTM disease. At the very least, our findings provide important factors for treatment continuation, as well as the safety and efficacy of NTM treatment in elderly patients with NTM disease.

## 5. Conclusions

Our current data suggested that age may not be an obstacle for receiving the benefits of the treatment of NTM disease in elderly patients. Furthermore, elderly patients without heart disease and ADL disability may have a better rate of continuing the NTM treatment, suggesting a better outcome for this field in an aging society. Selecting standard regimens and not modifying the NTM treatment is important for the maximum treatment efficacy.

## Figures and Tables

**Table 1:** Baseline characteristics of treated elder patients with pulmonary nontuberculous mycobacterial disease.

Characteristics	Treated Elder n = 40
Age (years old)	79.1 (0.8)
Gender (female)	26 (65.0)
BMI (kg/m^2^)	20.0 (3.6)
Patient With Smoking History	14 (41.2)
Patient With Habit of Alcohol	8 (25.8)
Comorbidities	24 (60.0)
Respiratory Disease	18 (45.0)
Malignancy	9 (22.5)
Heart Disease	7 (17.5)
Patient with ADL Disability	12 (30.0)
Symptoms	
Cough	23 (57.5)
Sputum	20 (50.0)
Hemoptysis	10 (25.0)
Dyspnea	2 (5.0)
Fever	5 (12.5)
Weight Loss	6 (15.0)
Radiological Type	
Fibrocavitary	14 (35.0)
Nodular Bronchiectatic	26 (65.0)
More Than Three Lobes Involved Based on CT	22 (55.0)
Diagnosed by Bronchofiberscopy	7 (17.5)
Prescribed Number of Antimycobacterial Drugs	
Three or More Drugs	28 (70.0)
Two Drugs	3 (7.5)
Single Drug	9 (22.5)
Use of Standard Regimens *	24 (60.0)

Abbreviations: ADL = activities of daily living; AMK = amikacin; BMI = body mass index; CAM = clarithromycin; CT = computed tomography; EB = ethambutol; INH = isoniazid; IPM/CS = imipenem/cilastatin; RFP = rifampicin. Data are presented as n (%) for categorical data or mean values (standard deviation) for numeric data. * Standard regimens were defined as combination of RFP, EB, and CAM for *M. avium* complex, combination of INH, RFP, and EB for *M. kansasii*, and combination of AMK, CAM, and IPM/CS for *M. abscessus*.

**Table 2:** Comparison of background characteristics of patients who changed/continued or discontinued the treatment.

Characteristics	Changed or Discontinued *n* = 18 (45.0%)	Continued *n* = 22 (55.0%)	*p*
Age (years old)	80.2 (4.9)	78.1 (4.7)	0.169
Gender (female)	10 (55.6)	16 (72.7)	0.257
BMI (kg/m^2^)	18.8 (2.6)	21.0 (4.1)	0.097
Patient With Smoking History	7 (46.7)	7 (36.8)	0.563
Patient With Habit of Alcohol	4 (26.7)	4 (18.2)	0.916
Comorbidities			
Respiratory Disease	10 (55.6)	8 (36.4)	0.225
Malignancy	5 (27.8)	4 (18.2)	0.705
Impaired Immunity	5 (27.8)	2 (9.1)	0.211
Heart Disease	6 (33.3)	1 (4.6)	0.033
Patient With ADL Disability	9 (50.0)	3 (13.6)	0.018
Symptoms			
Cough	10 (55.6)	13 (59.1)	0.822
Sputum	9 (44.4)	12 (54.6)	0.525
Hemoptysis	3 (16.7)	7 (31.8)	0.465
Dyspnea	0 (0.0)	2 (9.1)	0.492
Fever	4 (22.2)	1 (4.6)	0.155
Weight Loss	3 (16.7)	3 (13.)	1.000
Radiological Type			0.641
Fibrocavitary	7 (38.9)	7 (31.8)	
Nodular Bronchiectatic	11 (61.1)	15 (68.2)	
More Than Three Lobes Involved in CT	11 (61.1)	11 (50.0)	0.482
Diagnosed by Bronchofiberscopy	4 (22.2)	3 (13.6)	0.680
Prescribed Number of Antimycobacterial Drugs			0.583
Three or More Drugs	13 (72.2)	15 (68.2)	
Two Drugs	2 (11.1)	1 (4.6)	
Single Drug	3 (16.7)	6 (27.3)	
Standard Regimens *	11 (61.1)	13 (59.1)	0.897
Adverse Events Associated with Treatment	11 (61.1)	8 (36.4)	0.203

Abbreviations: ADL = activities of daily living; AMK = amikacin; BMI = body mass index; CAM = clarithromycin; CT = computed tomography; EB = ethambutol; INH = isoniazid; IPM/CS = imipenem/cilastatin; RFP = rifampicin. Data are presented as n (%) for categorical data or mean values (standard deviation) for numeric data. * Standard regimens were defined as combination of RFP, EB, and CAM for *M. avium* complex, combination of INH, RFP, and EB for *M. kansasii*, and combination of AMK, CAM, and IPM/CS for *M. abscessus*.

**Table 3:** Comparison of characteristics of patients based on adverse events associated with treatment.

Characteristics	AE *n* = 19 (47.5%)	No AE *n* = 21 (52.5%)	*p*
Age (years old)	77.7 (5.0)	80.2 (4.5)	0.116
Gender (female)	13 (68.4)	13 (61.9)	0.666
BMI (kg/m^2^)	19.5 (2.7)	20.4 (4.3)	0.341
Patient With Smoking History	5 (31.3)	9 (50)	0.315
Patient With Habit of Alcohol	3 (18.8)	5 (33.3)	0.433
Comorbidities			
Respiratory Disease	8 (42.1)	10 (47.6)	0.761
Malignancy	2 (10.5)	7 (33.3)	0.133
Impaired Immunity	4 (21.1)	3 (14.3)	0.689
Heart Disease	4 (21.1)	3 (14.3)	0.574
Patient With ADL Disability	8 (42.1)	4 (19.1)	0.170
Symptoms			
Cough	12 (63.2)	11 (52.4)	0.491
Sputum	10 (52.6)	10 (47.6)	0.752
Hemoptysis	6 (31.6)	4 (19.1)	0.473
Dyspnea	0 (0.0)	2 (9.5)	0.489
Fever	2 (10.5)	3 (14.3)	0.720
Weight Loss	3 (15.8)	3 (14.3)	1.000
Radiological Type			0.186
Fibrocavitary	9 (47.4)	5 (23.8)	
Nodular Bronchiectatic	10 (52.6)	16 (76.2)	
More Than Three Lobes Involved in CT	10 (52.6)	12 (57.1)	0.775
Diagnosed by Bronchofiberscopy	3 (15.8)	4 (19.1)	1.000
Prescribed Number of Antimycobacterial Drugs			0.258
Three or More Drugs	15 (79.0)	13 (61.9)	
Two Drugs	2 (10.5)	1 (4.8)	
Single Drug	2 (10.5)	7 (33.3)	
Standard Regimens *	13 (68.4)	11 (52.4)	0.349

Abbreviations: ADL = activities of daily living; AMK = amikacin; BMI = body mass index; CAM = clarithromycin; CT = computed tomography; EB = ethambutol; INH = isoniazid; IPM/CS = imipenem/cilastatin; RFP = rifampicin. Data are presented as n (%) for categorical data or mean values (standard deviation) for numeric data. * Standard regimens were defined as combination of RFP, EB, and CAM for *M. avium* complex, combination of INH, RFP, and EB for *M. kansasii*, and combination of AMK, CAM, and IPM/CS for *M. abscessus*.

**Table 4:** Comparison of characteristics between patients who had sputum conversion failed or achieved.

Characteristics	Sputum Culture Conversion Failed *n* = 12 (30.0%)	Sputum Culture Conversion Achieved *n* = 28 (70.0%)	*p*
Age (years old)	79.4 (5.5)	78.9 (4.7)	0.756
Gender (female)	9 (75.0)	17 (60.7)	0.385
BMI (kg/m^2^)	18.3 (3.7)	20.7 (3.4)	0.212
Patient With Smoking History	4 (40.0)	10 (41.7)	1.000
Patient With Habit of Alcohol	1 (11.1)	7 (31.8)	0.379
Comorbidities			
Respiratory Disease	6 (50.0)	12 (31.8)	0.173
Malignancy	2 (16.7)	7 (25.0)	0.697
Impaired Immunity	1 (8.3)	6 (21.4)	0.652
Heart Disease	3 (25.0)	4 (14.3)	0.410
Patient With ADL Disability	4 (33.3)	8 (28.6)	1.000
Symptoms			
Cough	7 (58.3)	16 (57.1)	0.944
Sputum	6 (50.0)	14 (50.0)	1.000
Hemoptysis	6 (50.0)	4 (14.3)	0.041
Dyspnea	1 (8.3)	1 (3.6)	0.515
Fever	1 (8.3)	4 (14.3)	1.000
Weight Loss	2 (16.7)	4 (14.3)	1.000
Radiological Type			0.484
Fibrocavitary	3 (25.0)	11 (39.3)	
Nodular Bronchiectatic	9 (75.0)	17 (60.7)	
More Than Three Lobes Involved in CT	7 (58.3)	15 (53.6)	0.077
Diagnosed by Bronchofiberscopy	0 (0.0)	7 (25.0)	0.081
Prescribed Number of Antimycobacterial Drugs			0.116
Three or More Drugs	6 (50.0)	22 (78.6)	
Two Drugs	2 (16.7)	1 (3.6)	
Single Drug	4 (33.3)	5 (17.9)	
Standard Regimens *	3 (25.0)	21 (75.0)	0.005

Abbreviations: ADL = activities of daily living; AMK = amikacin; BMI = body mass index; CAM = clarithromycin; CT = computed tomography; EB = ethambutol; INH = isoniazid; IPM/CS = imipenem/cilastatin; RFP = rifampicin. Data are presented as n (%) for categorical data or mean values (standard deviation) for numeric data. * Standard regimens were defined as combination of RFP, EB, and CAM for *M. avium* complex, combination of INH, RFP, and EB for *M. kansasii*, and combination of AMK, CAM, and IPM/CS for *M. abscessus*.

**Table 5:** Comparison of characteristics between patients who had radiologic findings improved or not.

Characteristics	Unchanged or Deteriorated *n* = 17 (42.5%)	Improved *n* = 23 (57.5%)	*p*
Age (years old)	78.5 (4.8)	79.5 (5.0)	0.556
Gender (Female)	14 (82.4)	12 (52.2)	0.092
BMI (kg/m^2^)	19.9 (4.7)	20.1 (2.8)	0.622
Patient With Smoking History	3 (17.6)	11 (47.8)	0.038
Patient With Habit of Alcohol	1 (5.9)	7 (30.4)	0.095
Comorbidities			
Respiratory Disease	7 (41.2)	11 (47.8)	0.676
Malignancy	4 (23.5)	5 (21.7)	0.893
Impaired Immunity	3 (17.6)	4 (17.4)	1.000
Heart Disease	4 (23.5)	3 (13.0)	0.432
Patient with ADL Disability	7 (41.2)	5 (21.7)	0.296
Symptoms			
Cough	12 (70.6)	11 (47.8)	0.202
Sputum	11 (64.7)	9 (39.1)	0.200
Hemoptysis	3 (17.6)	7 (30.4)	0.471
Dyspnea	1 (5.9)	1 (4.4)	1.000
Fever	2 (11.8)	3 (13.0)	1.000
Weight Loss	1 (5.9)	5 (21.7)	0.216
Radiological Type			0.041
Fibrocavitary	9 (52.9)	5 (21.7)	
Nodular Bronchiectatic	8 (47.1)	18 (78.3)	
More Than Three Lobes Involved in CT	11 (64.7)	11 (47.8)	0.289
Diagnosed by Bronchofiberscopy	2 (11.8)	5 (21.7)	0.677
Prescribed Number of Antimycobacterial Drugs			0.495
Three or More Drugs	10 (58.8)	18 (78.3)	
Two Drugs	2 (11.8)	1 (4.4)	
Single Drug	5 (29.4)	4 (17.4)	
Standard Regimens *	7 (41.2)	17 (73.9)	0.037

Abbreviations: ADL = activities of daily living; AMK = amikacin; BMI = body mass index; CAM = clarithromycin; CT = computed tomography; EB = ethambutol; INH = isoniazid; IPM/CS = imipenem/cilastatin; RFP = rifampicin. Data are presented as n (%) for categorical data or mean values (standard deviation) for numeric data. * Standard regimens were defined as combination of RFP, EB, and CAM for *M. avium* complex, combination of INH, RFP, and EB for *M. kansasii*, and combination of AMK, CAM, and IPM/CS for *M. abscessus*.
